# Inhibition of extracellular signal-regulated kinase 1/2 signaling has beneficial effects on skeletal muscle in a mouse model of Emery-Dreifuss muscular dystrophy caused by lamin A/C gene mutation

**DOI:** 10.1186/2044-5040-3-17

**Published:** 2013-07-01

**Authors:** Antoine Muchir, Young Jin Kim, Sarah A Reilly, Wei Wu, Jason C Choi, Howard J Worman

**Affiliations:** 1 Department of Medicine, College of Physicians and Surgeons, Columbia University, 630 West 168th Street, New York, NY 10032, USA; 2 Department of Pathology and Cell Biology, College of Physicians and Surgeons, Columbia University, New York, NY, USA; 3 Current address: Therapie des maladies du muscle strie, Institut de Myologie UM76 - UPMC Univ. Paris 6, U974 – Inserm, UMR7215 - CNRS G.H., F-75651 Paris, Cedex 13, France; 4 Current address: Stony Brook University School of Medicine, Stony Brook, NY 11794, USA; 5 Current address: Tufts University School of Medicine, Boston, MA 02111, USA

**Keywords:** Muscular dystrophy, Nuclear envelope, Lamin, Selumetinib, Mitogen-activated protein kinase

## Abstract

**Background:**

Autosomal Emery-Dreifuss muscular dystrophy is caused by mutations in the lamin A/C gene (*LMNA*) encoding A-type nuclear lamins, intermediate filament proteins of the nuclear envelope. Classically, the disease manifests as scapulo-humeroperoneal muscle wasting and weakness, early joint contractures and dilated cardiomyopathy with conduction block; however, move variable skeletal muscle involvement can be present. Previously, we demonstrated increased activity of extracellular signal-regulated kinase (ERK) 1/2 in hearts of *Lmna*^H222P/H222P^ mice, a model of autosomal Emery-Dreifuss muscular dystrophy, and that blocking its activation improved cardiac function. We therefore examined the role of ERK1/2 activity in skeletal muscle pathology.

**Methods:**

Sections of skeletal muscle from *Lmna*^H222P/H222P^ mice were stained with hematoxylin and eosin and histological analysis performed using light microscopy. ERK1/2 activity was assessed in mouse tissue and cultured cells by immunoblotting and real-time polymerase chain reaction to measure expression of downstream target genes. *Lmna*^H222P/H222P^ mice were treated with selumetinib, which blocks mitogen-activated protein kinase/extracellular signal-regulated kinase kinase 1/2 that activates ERK1/2, from 16 to 20 weeks of age to assess the effects of treatment on muscle histology, ERK1/2 activity and limb grip strength.

**Results:**

We detected enhanced activation of ERK1/2 in skeletal muscle of *Lmna*^H222P/H222P^ mice. Treatment with selumetinib ameliorated skeletal muscle histopathology and reduced serum creatine phosphokinase and aspartate aminotransferase activities. Selumetinib treatment also improved muscle function as assessed by *in vivo* grip strength testing.

**Conclusions:**

Our results show that ERK1/2 plays a role in the development of skeletal muscle pathology in *Lmna*^H222/H222P^ mice. They further provide the first evidence that a small molecule drug may be beneficial for skeletal muscle in autosomal Emery-Dreifuss muscular dystrophy.

## Background

Emery-Dreifuss muscular dystrophy (EDMD) is classically characterized clinically by a triad of: (1) slowly progressive muscle weakness and wasting in a scapulo-humeroperoneal distribution; (2) early contractures of the elbows, ankles, and posterior neck; and (3) dilated cardiomyopathy with conduction defects [1,2]. Contractures are usually the first clinical sign of the disease occurring in the first decade of life. During the second decade of life, the slowly progressive muscle weakness and wasting typically begin. At the end of the second decade, most patients develop evidence of cardiomyopathy [3-5].

EDMD can be inherited in a X-linked or autosomal fashion. X-linked EDMD is caused by mutations in *EMD* encoding emerin [6]. Emerin is an integral protein of the inner nuclear membrane [7,8]. The majority of autosomal dominant and less frequent recessive cases are caused by mutations in *LMNA*[9,10]. *LMNA* encodes two major somatic cell polypeptides, lamin A and lamin C, which are components of the nuclear lamina, a meshwork of intermediate filaments on the inner aspect of the inner nuclear membrane [11-14]. While the classical EDMD phenotype was first attributed to *EMD* and *LMNA* mutations, it is now apparent that the same mutations in these genes can cause dilated cardiomyopathy with more variable skeletal muscle involvement [6,9,15-21]. Intriguingly, *LMNA* mutations (different than those leading to myopathy) can also cause partial lipodystrophy, peripheral neuropathy, or accelerated aging disorders such as Hutchinson-Gilford progeria syndrome [22].

Despite the relatively recent advances in understanding the genetics of EDMD and related myopathies, the pathogenic mechanisms leading to striated muscle damage are only poorly understood. One useful small animal model to study pathogenesis and evaluate potential therapeutic interventions in autosomal EDMD is the *Lmna*^H222P/H222P^ mouse [23]. Starting at approximately 16 weeks, male *Lmna*^H222P/H222P^ develop progressive dystrophic pathology in several skeletal muscle groups. Later, they have progressive accumulation of connective tissue in skeletal muscle. *Lmna*^H222P/H222P^ mice also develop dilated cardiomyopathy with conduction system abnormalities and significant cardiac fibrosis.

We have previously shown that *Lmn*a^H222P/H222P^ mice have increased activity of the mitogen-activated protein kinase extracellular signal-regulated kinase (ERK) 1/2 in cardiac muscle [24]. This increased ERK1/2 activity occurs prior to the onset of overt tissue pathology, suggesting that it plays a primary pathogenic role. Treatment of *Lmn*a^H222P/H222P^ mice with drugs that inhibit mitogen-activated protein kinase/extracellular signal-regulated kinase kinase (MEK) 1/2, the kinase that activates ERK1/2, leads to improved left ventricular ejection fraction [25,26], decreased cardiac fibrosis [26,27] and prolonged survival [27]. While these results strongly suggest that abnormal ERK1/2 activation contributes to the development of cardiomyopathy in *Lmna*^H222P/H222P^ mice, its pathogenic role in affected skeletal muscles is unknown.

Based on our findings in heart, we hypothesize that abnormal activation of ERK1/2 is similarly involved in the pathogenesis of skeletal muscular dystrophy in the *Lmna*^H222P/H222P^ mouse model of EDMD. In the present study, we demonstrate increased activation of ERK1/2 in affected skeletal muscle these mice. We further show that treatment with the MEK1/2 inhibitor selumetinib ameliorates pathological changes and improves function. These results suggest that MEK1/2 inhibitors may be beneficial in treating both cardiac and skeletal muscle disease in patients with EDMD.

## Methods

### Mice

*Lmna*^H222P/H222P^ mice were bred and genotyped as previously described [23]. Mice were fed chow and housed in a disease-free barrier facility with 12 h/12 h light/dark cycles. The Institutional Animal Care and Use Committee at Columbia University Medical Center approved the use of animals and the study protocols.

### Drug treatment protocol and harvesting of muscle samples

Selumetinib (Selleck Chemicals) was dissolved in dimethyl sulfoxide (DMSO) (Sigma) at a concentration of 0.5 mg/mL to allow for intraperitoneal injections in mice. The placebo control consisted of the same volume of DMSO. Selumetinib was delivered at a dose of 1 mg/kg daily by intraperitoneal injection using a 27 5/8-gauge syringe starting when mice were 16 weeks of age and continuing until 20 weeks of age. At the end of the study, mice were sacrificed and hindlimb and diaphragm muscles dissected. Part of each dissected muscle was frozen in liquid nitrogen and stored at -80°C for biochemical analysis. The remaining muscle was rapidly frozen in isopentane pre-chilled by liquid nitrogen for cryostat sectioning.

### Histology

Frozen pieces of quadriceps femoris, diaphragm, and tibialis anterior were mounted in Tissue-Tek (Fisher Scientific) and 10 μm sections cut on a cryostat. Sections were stained with hematoxylin and eosin for histological analysis. Representative sections were photographed using a Microphot SA (Nikon) light microscope attached to a Spot RT Slide camera (Diagnostic Instruments). Images were processed using Adobe Photoshop CS (Adobe Systems).

### Osmotic shock of C2C12 cells stably expression wild-type and H222P lamin A

Generation of stable C2C12 cells expressing wild-type and H222P lamin A has been described previously [28]. These cells were maintained at 37°C with 5% CO_2_ and subcultured at approximately 60% to 70% confluence in Dulbecco’s modified Eagle’s medium supplemented with 10% fetal bovine serum (Invitrogen). To assess the impact of osmotic shock on the activation of ERK1/2, cells were treated with D-sorbitol (600 mM) for 1 h and proteins were harvested in RIPA extraction buffer (Cell Signaling Technology) as previously described [24].

### Quantitative real-time reverse transcription-polymerase chain reaction (RT-PCR)

Total RNA was extracted using the RNeasy isolation kit (Qiagen). Total RNA was used to synthesize cDNA using SuperScript First-strand Synthesis System (Invitrogen) according to the manufacturer’s instructions. For each replicate in each experiment, RNA from tissue samples of different animals was used. Primers were designed corresponding to mouse RNA sequences using Primer3 [29]. Real-time quantitative RT-PCR reactions contained HotStart-IT SYBR green qPCR Master Mix (Affymetrix), 200 nM of each primer and 0.2 μL of template in a reaction volume of 25 μL. Amplification was carried out using the ABI 7300 Real-time PCR System (Applied Biosystems). Relative levels of mRNA expression were calculated using the ΔΔC_T_ method [30] and individual expression values were normalized by comparison to *Gapdh* mRNA.

### Protein extraction and immunoblotting

Skeletal muscle was homogenized in RIPA extraction buffer (Cell Signaling Technology) as previously described [24]. Extracted proteins were separated by SDS-polyacrylamide gel electrophoresis, transferred to nitrocellulose membranes, and blotted with primary antibodies against ERK1/2 and phosphorylated ERK1/2 (Cell Signaling Technology). Secondary antibodies were horseradish peroxidate-conjugated (GE Healthcare). Recognized proteins were visualized by enhanced chemiluminescence (GE Healthcare). To quantify results, the immunoblots were scanned and band densities calculated using ImageJ64 (Applied Imaging). Signals obtained for phosphorylated ERK1/2 were normalized to those for total ERK1/2.

### Serum biochemistry

Serum was separated from mouse blood and stored at -80°C for 3 to 9 months until analyzed. Creatine phosphokinase (CPK) and aspartate aminotransferase (AST) activities were measured using an Analyst III Analyzer (Hemagen Diagnostics) in the Comparative Pathology Laboratory at Columbia University Medical Center. CPK and AST activities have been shown to be stable in rodent serum stored for up to 360 days at -70°C [31].

### Limb grip strength measurements

*Lmna*^H222P/H222P^ mice treated with DMSO or selumetinib were subjected to limb grip strength testing using a horizontally positioned grip strength meter (Bioseb). Mice were lowered by the tail towards the grid on the apparatus. Upon grasping the grid with their limbs, mice were pulled backward in the horizontal plane. The procedure was repeated consecutively three times and the peak tension of the three pulls was recorded as the grip strength value. Each animal was subjected to a total of two serial trials of three pulls each with 20 s of rest in between.

### Statistics

Values for real-time quantitative RT-PCR, scanned immunoblots, internalized nuclei, serum CPK and AST activities, and grip strength were compared using an unpaired Student t-tests. Values for Feret’s diameter were compared using two-way ANOVA. Statistical analyses were performed using Prism (GraphPad Software).

## Results and discussion

### Dystrophic skeletal muscle pathology in *Lmna*^H222P/H222P^ mice

**Figure 1:**
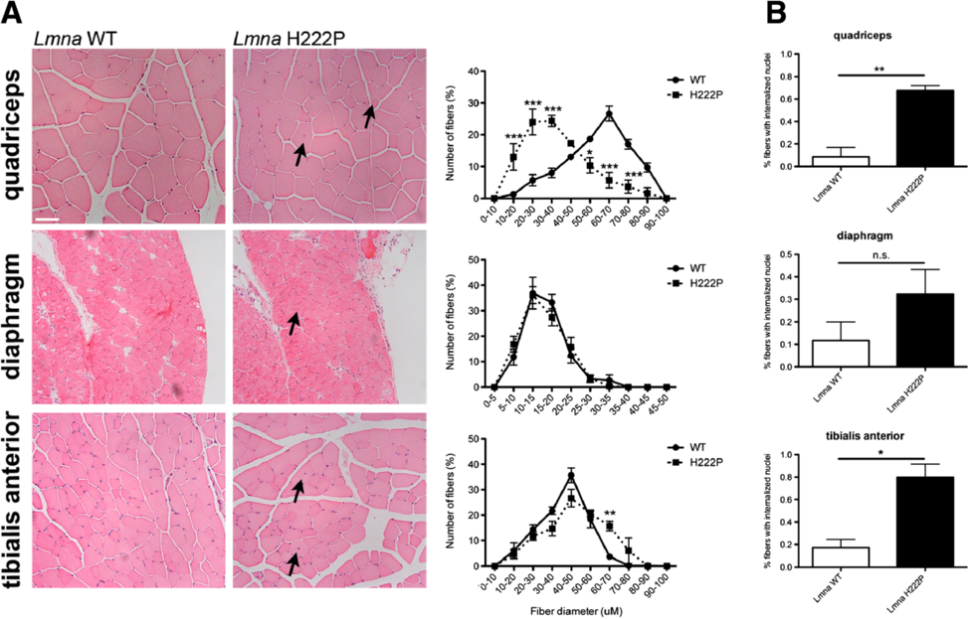
**Skeletal muscle pathology in *Lmna***^**H222P/H222P **^**mice.** (**A**) Representative micrographs of hematoxylin and eosin-stained sections of quadriceps, diaphragm, and tibialis anterior muscles from 20-week-old male *Lmna*^H222P/H222P^ mice (*Lmna* H222P). Similar sections from wild-type mice (*Lmna* WT) are shown for comparison. Scale bar = 50 mm. Arrows indicate internalized nuclei. To the right of each pair of micrographs, quantitative analyses of muscle fiber diameter (Feret’s diameter) are shown for wild-type (circles, sold line) and *Lmna*^H222P/H222P^ mice (squares, dashed line). Values are means ± SEM for *n* = 3 mice per group; **P <0.005, ***P <0.0005. (**B**) Bar graphs showing percentages of fibers in specified muscle groups with internalized nuclei. Values shown are means ± SEM for between 500 and 1,000 nuclei analyzed in tissue samples 3 per group; *P <0.05, **P <0.005, n.s. not significant.

Arimura *et al.*[23] previously reported progressive dystrophic changes in skeletal muscle starting at 16 weeks male *Lmna*^H222P/H222P^ mice. Their non-quantitative histopathological analysis included descriptions of a wide variation in fiber size, an increased number of atrophic, hypertrophic, and lobulated fibers, some regenerative fibers and a mention that some fibers had internalized nuclei. We therefore carefully quantified myofiber diameters and internalized nuclei in histological sections of quadriceps, diaphragm, and tibialis anterior muscle of male wild-type and *Lmna*^H222P/H222P^ mice at 20 weeks of age. Compared to wild-type mice, quadriceps and tibialis anterior from the *Lmna*^H222P/H222P^ mice exhibited a wider variation in fiber size (Figure 1A). In quadriceps, there was a clear shift towards smaller fiber diameters, consistent with the presence of greater numbers of atrophic and regenerative fibers. Both of these muscle groups also had an increased percentage of fibers with internal nuclei, which is observed during regeneration (Figure 1A,B). At this age, however, diaphragm did not show significant differences between *Lmna*^H222P/H222P^ and wild-type mice (Figure 1A,B).

### Abnormal ERK1/2 signaling in skeletal muscle of *Lmna*^H222P/H222P^ mice

**Figure 2:**
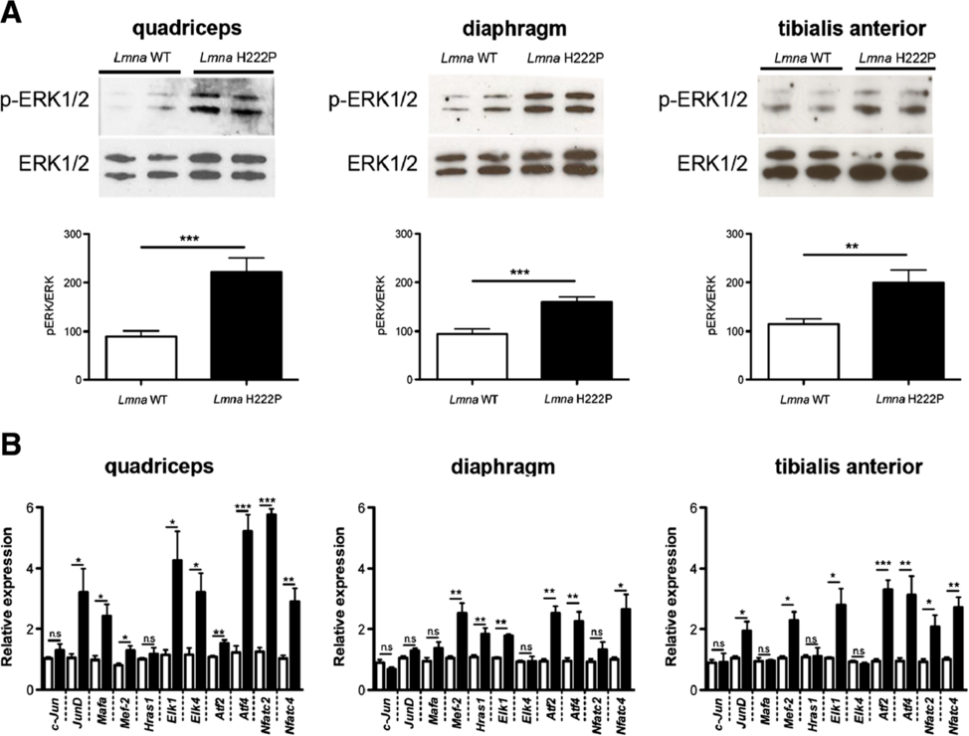
**Increased ERK1/2 activity in skeletal muscle of *Lmna***^**H222P/H222P **^**mice.** (**A**) Immunoblots showing phosphorylated ERK1/2 (p-ERK1/2) and total ERK1/2 in protein extracts from quadriceps, diaphragm, and tibialis anterior muscles of 20-week-old male wild-type (*Lmna* WT) and *Lmna*^H222P/H222P^ (*Lmna* H222P) mice. Each lane contains protein extracts from a different mouse. The bar graph shows means ± SEM values of pERK1/2 normalized to total ERK1/2 from scanned band densities of five immunoblots from *n* = 5 different mice per group. **P <0.005, ***P <0.0005. (**B**) Differential expression of 11 selected genes in the ERK1/2 signaling pathway analyzed using real-time quantitative RT-PCR in quadriceps, diaphragm, and tibialis anterior muscles of 20-week-old male wild-type and *Lmna*^H222P/H222P^ mice. White bars show relative RNA expression levels in skeletal muscles from wild-type mice *Lmna*^+/+^ mice and black bars in skeletal muscles from *Lmna*^H222P/H222P^ mice. Values are means ± SEM for *n* = 5 mice per group; the real-time quantitative RT-PCR was performed in triplicate on each different RNA sample. *P <0.05, **P <0.005, ***P <0.0005, n.s. not significant.

Hearts of *Lmna*^H222P/H222P^ mice and human subjects with autosomal EDMD have increased activity of ERK1/2, which likely plays a role in pathogenesis of cardiomyopathy [24-27]. We hypothesized that a similar increased activation of this signaling pathway occurs in skeletal muscle. We therefore examined ERK1/2 activity in skeletal muscle from 20-week-old male *Lmna*^H222P/H222P^ mice. Immunoblotting with antibody against phosphorylated (activated) ERK1/2 demonstrated a two-fold increase in activity in quadriceps, diaphragm, and tibialis anterior of *Lmna*^H222P/H222P^ mice compared to wild type mice (Figure 2A). We then used quantitative real-time PCR to measure expression of downstream ERK1/2 target genes, several of which are members of the ETS family of transcription factors that are phosphorylated by ERK1/2 and positively autoregulate their transcriptional activity [24,32,33]. Of 11 targets genes assessed, we detected significantly increased expression of mRNAs for nine in quadriceps, six in diaphragm, and seven in tibialis anterior of *Lmna*^H222P/H222P^ mice compared to wild-type controls (Figure 2B). Among these, *Mef-2*, *Elk1*, *Atf2*, *Atf4*, and *Nfatc-4* showed significantly increased expression in the three skeletal muscles examined. These data demonstrate that ERK1/2 is hyperactivated in the skeletal muscles of *Lmna*^H222P/H222P^ mice. Increased ERK1/2 activation in diaphragm at an age before there is any detectable histological abnormalities is consistent with its increased activity in heart prior to the onset of detectable pathological signs of cardiomyopathy [24]. This suggests that increased ERK1/2 signaling is involved in the pathogenesis of dystrophic skeletal muscle pathology.

### Stress-induced activation of ERK1/2 in cultured myoblasts stably expressing H222P lamin A

**Figure 3:**
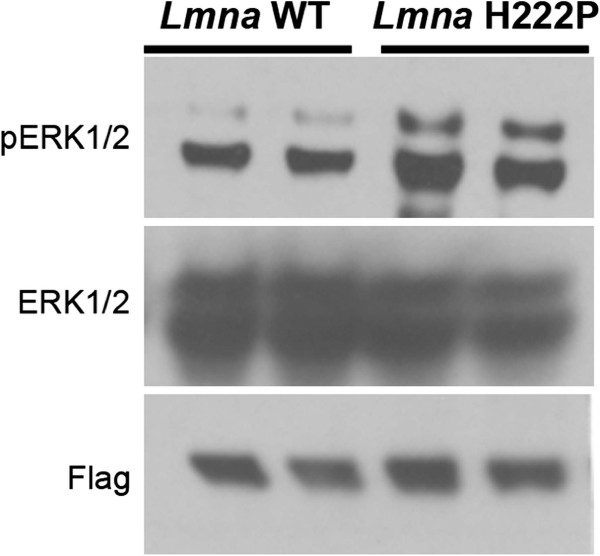
**Cultured myoblasts expressing H222P lamin A have greater ERK1/2 activity after osmotic shock than those expressing wild-type lamin A.** One hour after osmotic shock with D-sorbitol, protein extracts from C2C12 cells stably expressing flag-tagged wild-type lamin A (*Lmna* WT) and flag-tagged H222P lamin A (*Lmna* H222P) were analyzed by immunoblotting. Blots were probed with antibodies against phosphorylated ERK1/2 (pERK1/2), total ERK1/2 (ERK1/2) and Flag. The immunoblot shown is representative of three separately performed experiments.

We have previously shown that transient transfection of C2C12 mouse myoblasts with cDNA encoding H222P prelamin A or other variants associated with striated muscle disease have increased ERK1/2 activity compared to those transfected with a cDNA encoding wild-type prelamin A [24]. However, stably transfected C2C12 cells expressing H222P lamin A do not have increased ERK1/2 activity at baseline but do after glucose depravation or treatment with 5-aminoimidazole-4-carboxyamide ribonucleoside [28]. This led us to hypothesize that physiological stress, such as that associated with manipulations necessary for transient transfection or induced by altered energy metabolism, is necessary to increase ERK1/2 activity in myoblasts expressing lamin A variants. We further tested this hypothesis by subjecting the same cells stably expressing lamin A H222P that do not have baseline elevation in ERK1/2 [28] to osmotic shock. One hour after an osmotic shock with 600 mM D-sorbitol, cells expressing flag-tagged H222P lamin A had a greater activity of ERK1/2 compared to those expressing flag-tagged wild-type lamin A (Figure 3). This result provided additional support for a model in which alterations in the nuclear lamina associated with striated muscle disease lead to abnormalities in the activities of cellular stress-responsive signaling pathways [24,34,35]. The requirement of a stress to hyperactivate ERK1/2 in cells expressing the H222P lamin A may also at least in part explain why striated muscle, a tissue repeatedly under mechanical strain, is preferentially affected by *LMNA* mutations generating certain A-type lamin variants.

### Blocking ERK1/2 activity with selumetinib has beneficial effects on skeletal muscle in *Lmna*^H222P/H222P^ mice

**Figure 4:**
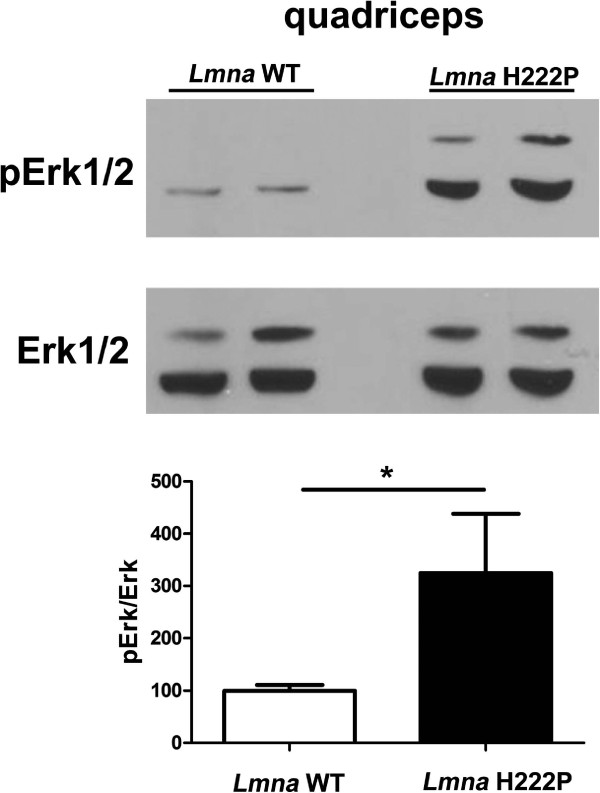
**Increased ERK1/2 activity in skeletal muscle of *Lmna***^**H222P/H222P **^**mice at 16 weeks of age.** Immunoblot showing phosphorylated ERK1/2 (p-ERK1/2) and total ERK1/2 in protein extracts from quadriceps of 16-week-old male wild-type (*Lmna* WT) and *Lmna*^H222P/H222P^ (*Lmna* H222P) mice. Each lane contains protein extracts from a different mouse. The bar graph shows means ± SEM values of pERK1/2 normalized to total ERK1/2 from scanned band densities of three immunoblots from *n* = 3 different mice per group. *P <0.05.

Given the enhanced ERK1/2 activity in skeletal muscle of *Lmna*^H222P/H222P^ mice that develop muscular dystrophy, we hypothesized that it may contribute to pathology. To test this hypothesis, we set up experiments to determine if inhibiting ERK1/2 signaling would prevent the progression of muscular dystrophy. At 16 weeks of age, ERK1/2 activity was elevated in quadriceps muscle of *Lmna*^H222P/H222P^ mice compared to wild-type mice, as assessed by immunoblotting with antibody against phosphorylated kinase (Figure 4).

**Figure 5:**
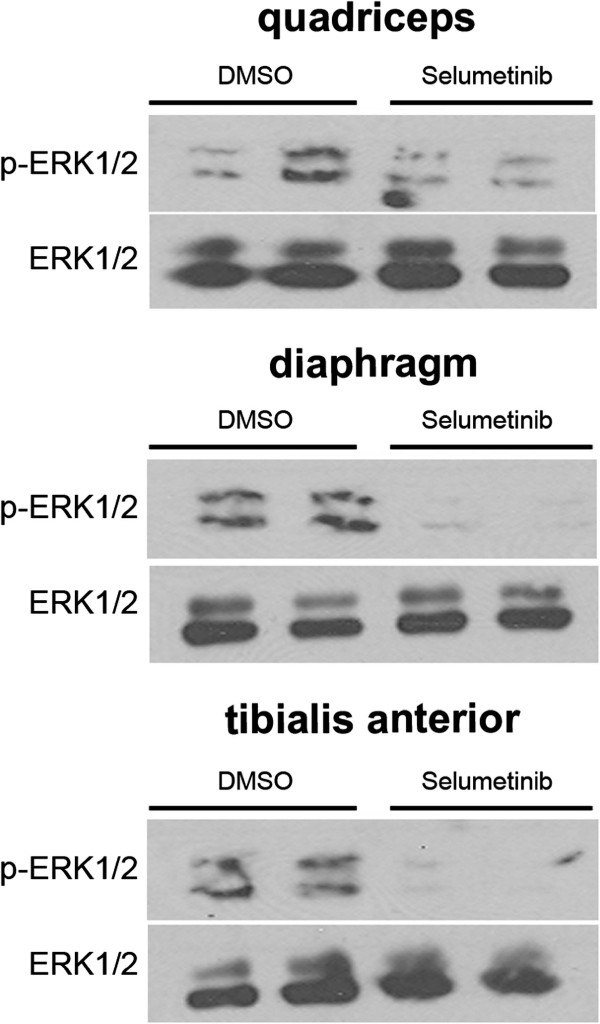
**Selumetinib inhibits ERK1/2 phosphorylation in skeletal muscles from *Lmna***^**H222P/H222P **^**mice.** Representative immunoblots using antibodies against phosphorylated ERK1/2 (pERK1/2) and total ERK1/2 (ERK1/2) to probe proteins extracts from quadriceps, diaphragm, and tibialis anterior from 20-week-old male *Lmna*^H222P/H222P^ mice treated with selumetinib or DMSO for 4 weeks. The immunoblot shown is representative of three separately performed experiments.

We administered the MEK1/2 inhibitor selumetinib to male *Lmna*^H222P/H222P^ mice by giving daily intraperitoneal injections (1 mg/kg) starting at 16 weeks of age. After 4 weeks of treatment, the mice had reduced phosphorylated ERK1/2 in quadriceps, tibialis anterior, and diaphragm compared to placebo-treated mice. This demonstrated that systemically administered selumetinib inhibited ERK1/2 signaling in skeletal muscle (Figure 5).

**Figure 6:**
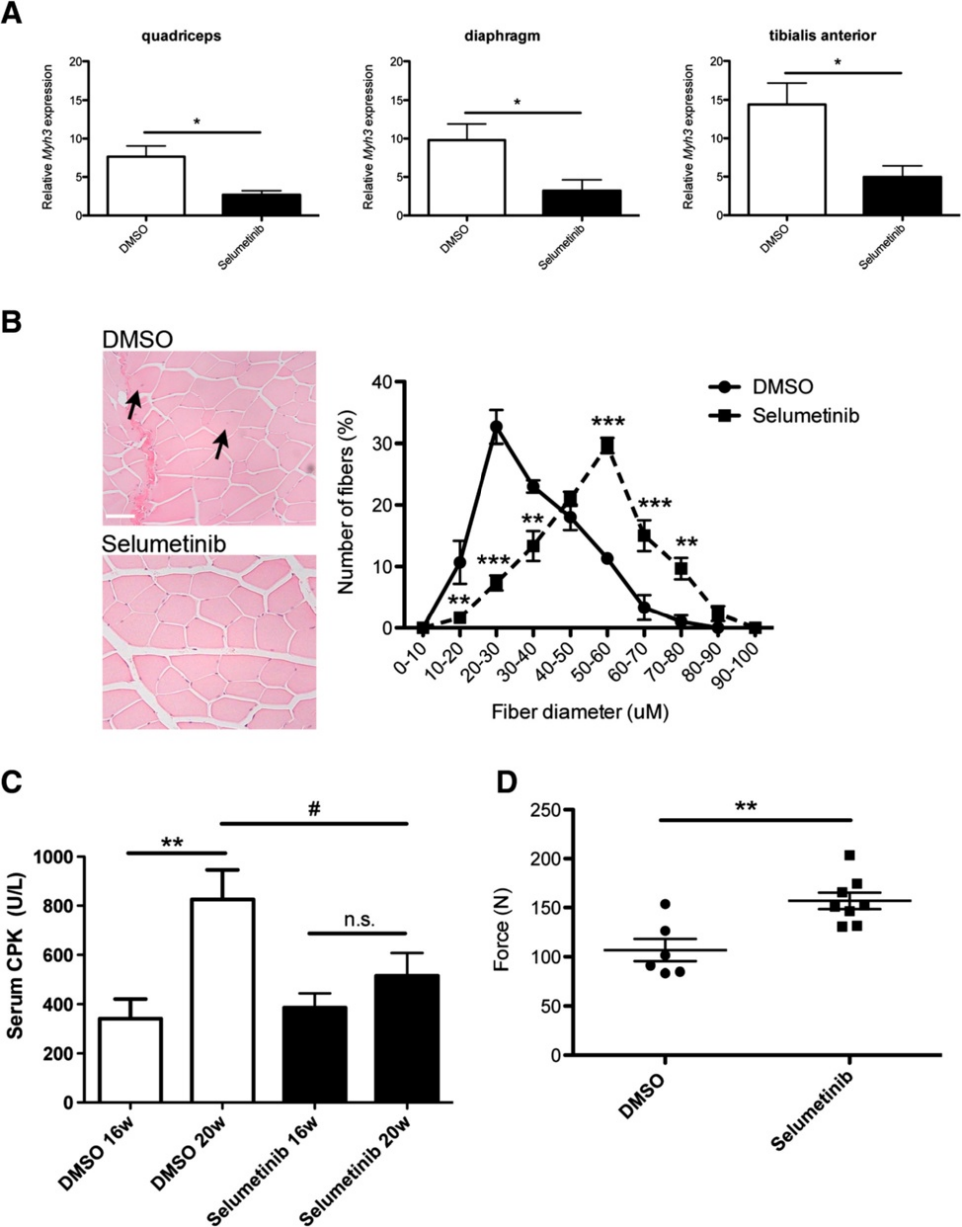
**Selumetinib from 16 to 20 weeks of age improves skeletal muscle pathology and function in *Lmna***^**H222P/H222P **^**mice.** (**A**) Expression of *Myh3* in *Lmna*^H222P/H222P^ mice measured using real-time quantitative RT-PCR. White bars show relative RNA expression levels in skeletal muscles of DMSO-treated (white bars) and selumetinib-treated (black bars) mice. Values are means ± SEM for *n* = 5 mice per group; the real time RT-PCR was performed in triplicate with the different RNA sample; *P <0.05. (**B**) Representative micrographs of hematoxylin and eosin-stained sections of quadriceps from 20-week-old male *Lmna*^H222P/H222P^ mice (*Lmna* H222P) treated for 4 weeks with DMSO or selumetinib. Scale bar = 50 mm. Arrows indicate internalized nuclei. To the right of the micrographs, quantitative analyses of muscle fiber diameter (Feret’s diameter) are shown for mice treated with DMSO (circles, solid line) and selumetinib (squares, sold line). Values are means ± SEM for *n* = 3 mice per group; **P <0.005, ***P <0.0005. (**C**) Serum CPK activities in *Lmna*^H222P/H222P^ mice at 16 weeks (16 W) and 20 weeks (20 W) of age that were treated with DMSO (white bars) or selumetinib (black bars). Values are means ± SEM for *n* = 7 DMSO-treated mice and *n* = 15 selumetinib-treated mice; **P <0.005, n.s. not significant, ^#^*P* <0.05. (**D**) Grip strength (force) in Newtons (N) in *Lmna*^H222P/H222P^ mice at 20 weeks of age that were treated with DMSO (circles) (*n*=6) or selumetinib (squares) (*n*=8). Each circle and square represents a measurement from an individual mouse; the longer horizontal bar are means and the shorter horizontal bars ± SEM; **P <0.05.

Following 4 weeks of treatment with selumetinib, there was significantly reduced expression of embryonic myosin heavy chain (*Myh3*) mRNA in quadriceps, diaphragm, and tibialis anterior of *Lmna*^H222P/H222P^ mice (Figure 6A). This represented a partial reversal of embryonic myosin expression that typically occurs in dystrophic muscle [36,37]. While quadriceps from DMSO-treated mice had 0.52% fibers (4/772 from three mice) with internalized nuclei (Figure 6A, arrows), there were none detected in 571 fibers from three mice in the selumetinib-treated mice (and 1/604 fibers from three wild-type mice as shown in Figure 1B). DMSO treatment did not impact myofiber diameter compared to untreated *Lmna*^H222P/H222P^ mice; however, mice treated with selumetinib had a greater myofiber diameter in quadriceps compared to those treated with DMSO (Figure 6B). Between 16 and 20 weeks of age, there was a significant increase in serum CPK activity in *Lmna*^H222P/H222P^ mice treated with DMSO; however, CPK activity did not significantly increase in the mice that received selumetinib and at 20 weeks it was significantly lower than in those that received DMSO (Figure 6C). Mean serum AST activity was also significantly lower in the selumetinib-treated mice compared to the placebo-treated mice at 20 weeks of age (data not shown). To determine if selumetinib improved skeletal muscle function in *Lmna*^H222P/H222P^ mice, we evaluated limb grip strength. At 20 weeks of age, mean grip strength was significantly greater in selumetinib-treated *Lmna*^H222P/H222P^ mice than in DMSO-treated mice (Figure 6D). Hence, selumetinib improved skeletal muscle dystrophic pathology and improved function in *Lmna*^H222P/H222P^ mice.

## Conclusions

We have shown increased activity of ERK1/2 in skeletal muscle of the *Lmna*^H222P/H222P^ mouse model of autosomal EDMD and that blocking its activity ameliorates pathology. These results are in accordance with a growing body of research providing evidence that alterations in various cellular signaling pathways, including ERK1/2, are involved in the pathogenesis of muscular dystrophy [38]. In addition to autosomal EDMD, ERK1/2 has been implicated as contributing to skeletal or cardiac muscle pathology in *mdx*[39-41], γ-sarcoglycan-deficient [42,43], and *Lama2*^Dy-w^[44] mice, respective small animal models of Duchenne, limb girdle type 2C, and a form of congenital muscular dystrophy. ERK1/2 activity is also abnormally increased in hearts of mice with emerin deficiency, which is the genetic alteration in X-linked EDMD [45].

Blocking increased ERK1/2 signaling activity with selumetinib had beneficial effects on skeletal muscle function in *Lmna*^H222P/H222P^ mice. Previously, we obtained similar results with respect to the cardiomyopathy that occurs in these mice [24-27]. In a human clinical trial, selumetinib has been reported to promote muscle gain in patients with cholangiocarcinoma [46]. As oral selumetinib and other orally bioavailable MEK1/2 inhibitors with encouraging safety profiles are currently in clinical development for other indications [47,48], pilot trials in patients with EDMD and possibly other muscular dystrophies should be considered.

## Abbreviations

AST: Aspartate aminotransferase; CPK: Creatine phosphokinase; DMSO: Dimethyl sulfoxide; EDMD: Emery-Dreifuss muscular dystrophy; ERK: Extracellular signal-regulated kinase; LMNA: Lamin A/C gene; MEK: Mitogen-activated protein kinase/extracellular signal-regulated kinase kinase; RT-PCR: Reverse transcription-polymerase chain reaction.

## Competing interests

AM and HJW are inventors on a pending patent application (PCT/US09/42614) on methods for treating and/or preventing cardiomyopathies by ERK and JNK inhibition filed by the Trustees of Columbia University in the City of New York.

## Authors’ contributions

AM conceived of the study, bred mice, treated mice with drugs, carried out experiments measuring ERK1/2 activity in mouse tissue and cells, assessed skeletal muscle pathology and grip strength in mice, and drafted the manuscript. YJK carried out experiments on measuring ERK1/2 activity in mouse tissue and cells and assessing skeletal muscle pathology in mice. SAR assisted with isolating skeletal muscle form mice and participated in experiments measuring ERK1/2 activity in mouse tissue. WW bred mice, drew blood from mice, and assisted in treating mice with drugs. JCC generated stable cell lines expressing H222P and wild type lamin A. HJW helped conceive the study, supervised and coordinated all of the research, and wrote the final manuscript. All of the authors read and approved the final manuscript.
